# A study on the diagnosis of the *Helicobacter pylori* coccoid form with artificial intelligence technology

**DOI:** 10.3389/fmicb.2022.1008346

**Published:** 2022-10-28

**Authors:** Zishao Zhong, Xin Wang, Jianmin Li, Beiping Zhang, Lijuan Yan, Shuchang Xu, Guangxia Chen, Hengjun Gao

**Affiliations:** ^1^School of Medicine, Institute of Digestive Disease, Tongji University, Shanghai, China; ^2^China Center for Helicobacter pylori Molecular Medicine, Shanghai, China; ^3^The Second Affiliated Hospital of Guangzhou University of Chinese Medicine, Guangzhou, China; ^4^Tongji Hospital, School of Medicine, Tongji University, Shanghai, China; ^5^School of Chemical Engineering and Technology, China University of Mining and Technology, Xuzhou, China; ^6^Unicom Guangdong Industrial Internet Co., Ltd, Guangzhou, China; ^7^Department of Gastroenterology, Xuzhou Municipal Hospital Affiliated to Xuzhou Medical University, Xuzhou, China; ^8^National Engineering Center for Biochips, Shanghai, China

**Keywords:** *Helicobacter pylori*, coccoid form, artificial intelligence technology, deep learning, diagnosis

## Abstract

**Background:**

*Helicobacter pylori (H. pylori)* is an important pathogenic microorganism that causes gastric cancer, peptic ulcers and dyspepsia, and infects more than half of the world’s population. Eradicating *H. pylori* is the most effective means to prevent and treat these diseases. *H. pylori* coccoid form (HPCF) causes refractory *H. pylori* infection and should be given more attention in infection management. However, manual HPCF recognition on slides is time-consuming and labor-intensive and depends on experienced pathologists; thus, HPCF diagnosis is rarely performed and often overlooked. Therefore, simple HPCF diagnostic methods need to be developed.

**Materials and methods:**

We manually labeled 4,547 images from anonymized paraffin-embedded samples in the China Center for *H. pylori* Molecular Medicine (*CCHpMM*, Shanghai), followed by training and optimizing the Faster R-CNN and YOLO v5 models to identify HPCF. Mean average precision (mAP) was applied to evaluate and select the model. The artificial intelligence (AI) model interpretation results were compared with those of the pathologists with senior, intermediate, and junior experience levels, using the mean absolute error (MAE) of the coccoid rate as an evaluation metric.

**Results:**

For the HPCF detection task, the YOLO v5 model was superior to the Faster R-CNN model (0.688 vs. 0.568, mean average precision, mAP); the optimized YOLO v5 model had a better performance (0.803 mAP). The MAE of the optimized YOLO v5 model (3.25 MAE) was superior to that of junior pathologists (4.14 MAE, *p* < 0.05), no worse than intermediate pathologists (3.40 MAE, *p* > 0.05), and equivalent to a senior pathologist (3.07 MAE, *p* > 0.05).

**Conclusion:**

HPCF identification using AI has the advantage of high accuracy and efficiency with the potential to assist or replace pathologists in clinical practice for HPCF identification.

## Introduction

*Helicobacter pylori* is a gram-negative bacterium that colonizes the human gastric mucosa (Schistosomes, 1994; Hooi et al., 2017). It commonly causes infectious disease, with the global prevalence approaching half of the population (Sugano et al., 2015; Hooi et al., 2017). *H. pylori* can cause various gastric diseases, including chronic gastritis, peptic ulcer, and gastric mucosa-associated tissue lymphoma (Schistosomes, 1994, Sultan Qaboos University, 2007). It is classified as a Group I carcinogen by the International Agency for Research on Cancer (IARC) (Schistosomes, 1994) and a principal cause of intestinal-type gastric cancer (IARC Working Group on the Evaluation of Carcinogenic Risks to Humans, 1989). Eradicating this bacterium is critical to reducing the risk of gastric cancer and other diseases.

*Helicobacter pylori* coccoid form (HPCF) is a significant cause of refractory *H. pylori* and is generally ignored in clinical practice (Ierardi et al., 2020; Krzyzek and Grande, 2020; Shah et al., 2021). HPCF is an adaptation to a non-optimal environment in which *H. pylori* transforms from bacillar to coccoid form in response to adverse growth conditions, including nutrient deficiencies, altered oxygen concentrations, elevated temperatures, and particularly sub-lethal antibiotic doses (Cellini et al., 2008; Kadkhodaei et al., 2020). And HPCF, a viable but non-culturable (VBNC) *H. pylori* form (Ozcakir, 2007), can result in cultivation failure, leading to false negatives in *H. pylori* diagnosis (Sarem and Corti, 2016). When *H. pylori* is in a coccoid state, it increases tolerance to the higher concentrations of antibiotics, favouring their survival (Tutelyan et al., 2015; Reshetnyak and Reshetnyak, 2017). And it causes non-response to antibiotics, resulting ineffective eradication treatment (Gladyshev et al., 2022). Most physicians do not recognize HPCF and administer other antibiotics to patients after a failed first intervention, which have no effect and aggravate resistance to more antibiotics. Therefore, according to the personalized assessment requirement, doctors need to recognize HPCF and discontinue antibiotics immediately until *H. pylori* returns to the bacillar form. Besides, HPCF has a low metabolism but does not lose its virulence, so toxic substances accumulate (Loke et al., 2016). Hence coccoid forms are a threat to the effectiveness of therapy and timely HPCF detection is a key component of personalized assessment before refractory *H. pylori* treatment.

The current clinical practice of identifying HPCF involves a pathologist observing stained *H. pylori* on gastric mucosa slices under a microscope and selecting a random field of view to estimate the HPCF proportion. However, the current approach is time-consuming, labor-intensive, and difficult to popularize. Additionally, diagnosis is inconsistent among pathologists owing to experience differences. Furthermore, as the field of view under the microscope is limited, the manual slide reading is randomly selected, leading to diagnosis limitations, randomness, and bias. Improving the efficiency and accuracy of HPCF diagnosis and reducing the difficulty in reading slides are essential for clinicians to provide more effective treatment options and promote this diagnostic method.

Artificial intelligence (AI) technology has facilitated the rapid development of object detection algorithms, which can be utilized for HPCF diagnosis to solve the problems caused by manual reading. The purpose of object detection is to find all objects of interest in an image and determine their positions and sizes (Mane et al., 2008). An object detection diagnostic model plays an essential role in the diagnosis and prognosis of disease, including bone marrow cell automatic detection (Tayebi et al., 2022), tumor region identification in breast cancer samples (Joseph et al., 2019), and tongue cancer diagnosis (Heo et al., 2022). Therefore, object detection techniques may potentially contribute to solving HPCF diagnosis drawbacks.

The aim of this study was to assess HPCF identification using two representative models: the one-stage model YOLO v5 and two-stage model Faster-RCNN (Ren et al., 2017; Jocher et al., 2022). We collected clinical *H. pylori*-positive gastric mucosa samples, performed *H. pylori* immunochemical staining, labeled the images based on manual morphological classification, trained the model, and conducted object recognition with evaluation. The use of AI in diagnosis could increase the accuracy of recognizing HPCF and reduce manual reading errors, making HPCF detection universally popularized. Additionally, these efficient, rapid, and convenient methods could enable HPCF to be diagnosed immediately in refractory *H. pylori* infections to ensure that coping strategies are found.

## Materials and methods

### Data collection and immunocytochemistry

Clinical samples were obtained from the biobank of the China Center for *H. pylori* Molecular Medicine (*CCHpMM*, Shanghai, China). We randomly selected paraffin-embedded gastric mucosa samples from 34 patients. Ethical approval was not required for this study as the samples were completely anonymous, and no detailed personal information was obtained.

Immunochemical staining was performed on the obtained samples. First, we preprocessed the tissue. The gastric mucosa samples placed in 4% paraformaldehyde were fixed for 24 h and then made into paraffin sections by conventional dehydration, embedding, and sectioning. Second, staining was applied to enhance differentiation between the gastric cells and *H. pylori.* Generally, staining included eight steps: dewaxing and hydration, antigen repair, endogenous peroxidase blocking, primary antibody addition, enzyme-labeled polymer addition, color development, re-staining, dehydration, transparency, and sealing. The dyed samples were then scanned using an Aperio Scanscope (Aperio XT, Leica, Germany) at 40× magnification and acquired in.tiff file format.

### Slicing and labeling

The full image was cut into small 500 × 500 pixel images for labeling *H. pylori* morphology. All stained *H. pylori* in the images were labeled according to morphological characteristics (Table 1) as bacillar, coccoid, cross-section, transitional, and cluster. Each bacterium was labeled individually with a square box form in Colabeler v2.0.4 software by two well-trained pathologists with 8 years of clinical experience in *H. pylori* pathologic diagnosis. When a disagreement occurred concerning labeling, the final decision was made by a third senior pathologist after joint discussion. The labeled image was then generated as an XML file containing the marker coordinate, marker type, and image information to construct the VOC 2007 data file.

**Table 1:** Examples and characterization of five *Helicobacter pylori* morphology categories for distinguishing types and bacteria labeling.

Category name	Legend	Characterization
Bacillar	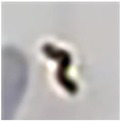	The normal spiral form of *H. pylori*, usually being longitudinal, bent, and curled
Coccoid	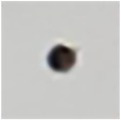	Rounded shape, the diameter is 1/3–1/2 the length of the bacterium
Cross section	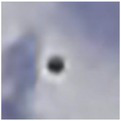	The circular cross-section when the bacillus is held upright
Transitional	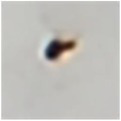	The stubby and irregular state presented by *H. pylori* as it transforms from bacillar to coccoid, not the coccoid
Cluster	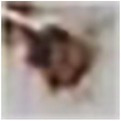	Multiple clustered bacteria, and we could not accurately label each bacterium individually

### Model frameworks selection and training

Faster R-CNN and YOLO v5 algorithms were chosen for object detection to select the one with better performance and perform downstream tasks, optimizing the model.

#### Faster R-CNN

Faster R-CNN is an end-to-end CNN object detection model with a two-stage target detection algorithm. The framework is divided into four phases: Conv layers, Region Proposal Networks, Roi Pooling, and Classifier. This algorithm is characterized by high detection accuracy and relatively slow detection speed.

#### YOLO v5

YOLO is a fast and compact open-source object detection model with better performance for the same size and better stability than other networks. It is the first end-to-end neural network to predict an object’s class and bounding box. YOLO v5, one of the most advanced object detection techniques, consists of four components: Input, Backone, Neck, and Prediction. YOLO v5 offers a wider variety of data enhancements to images on the input side, including mosaic data enhancement and adaptive image scaling, than traditional target detection models.

#### Dataset partitioning

We randomly divided the dataset into the train, validation, and test sets at a ratio of 6: 2: 2. The train and validation sets were used to train models and the test set was used to evaluate model performance. We judged the model’s training epochs according to the training curve. We found that as the epoch increased to 59, the train and validation sets’ loss decreased slowly and stabilized, indicating that the deep learning had reached saturation.

### Optimized measures

#### Data enhancement method

The label types showed a data imbalance in our study. To address this, we adopted a data enhancement copy-paste method by cropping targets from the positive data and randomly pasting them onto the negative sample (background information in the negative sample favors false positive suppression). The crop’s target frame may differ significantly in color from the randomly selected negative image. To reduce the “disharmony,” the crop’s target is toned to the negative background style using a stain normalization algorithm.

#### Model structure optimization

**Figure 1:**
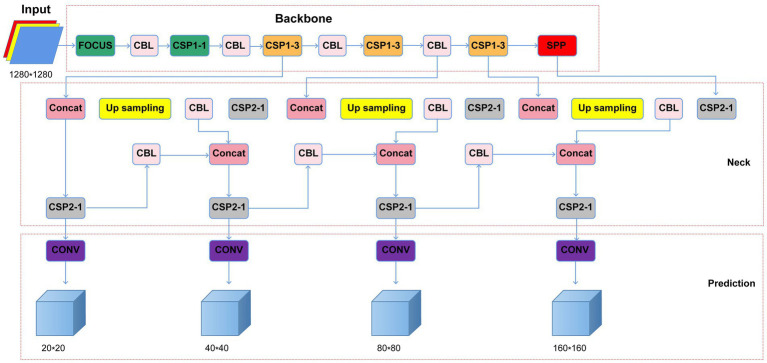
Optimized YOLO v5 network structure (CBL, Conv+Bn + Leaky-relu; CSP, cross stage partial network; Concat, concatenate function; Conv, convolutional layer; SPP, spatial pyramid pooling).

Considering that *H. pylori* occupies a relatively small part of the image, background iterative accumulation creates a large amount of redundant information accumulated during convolution, losing some smaller targets and resulting in low detection accuracy. Therefore, we applied coordinate attention (CA) between the backbone feature extraction network CSPDarkNet53 convolutional layers to improve the model’s object detection objects’ feature information extraction (Chao et al., 2021; Fang et al., 2022). CA changed the 10-layer network in the original YOLO v5 algorithm feature extraction to a 13-layer network and set the input image size to 1,280 × 1,280 scale. Furthermore, we added a 160 × 160 inspection scale to the original three-scale YOLO v5 inspection layer, expanding it to a four-scale inspection. Finally, we optimized the box anchor parameters, adding three anchor boxes for detecting small objects’ dimensions, including (5, 6, 8, 14, 15, and 11). The optimized YOLO v5 network structure is shown in Figure 1.

### Evaluation

To evaluate our model, we evaluated precision (P), recall (R), and mean average precision (mAP) using the following equations:


(1) $$\mathrm{Recall}=TP/\left(TP+FN\right)$$

(2) $$\mathrm{Precision}=TP/\left(TP+FP\right)$$

TP, FP, and FN in the above equations are defined as:

TP (True Positive): The number of positive and predicted positive samples.

FN (False Negative): The number of positive samples predicted as negative samples.

FP (False Positive): The number of negative samples predicted as positive samples.

For the object detection model’s comprehensive clinical evaluation, the samples’ HPCF percentages were evaluated by nine pathologists who were blinded to sample labeling. We invited three junior, three intermediate, and three senior pathologists with 5, 8, and 10 years of pathological work experience, respectively, to independently read the slice and confirm the coccoid percentage. Mean absolute error (MAE) helps reflect the forecast value error’s actual situation. The formula is as follows:


(3) $$MAE=\frac{1}{m}\overset{m}{\underset{1}{\sum }}|h-y|$$

Where m, h, and y represent the number of uncut sample sheets in the test set, gold standard (HPCF percent obtained by software labeling manually), and HPCF percentage of model detection or manual reading, respectively. We used the manual labeling results as the gold standard, and the model detection and manual reading results as the true values; the higher the error, the higher the MAE. All statistical analyses were performed using Python, version 3.8.5.

## Results

### Dataset generation

We obtained 4,547 sub-images for model training and testing. The various *H. pylori* morphology label distribution manually marked by the pathologists is shown in Supplementary Appendix S1.

### Faster R-CNN vs. YOLO v5

**Figure 2:**
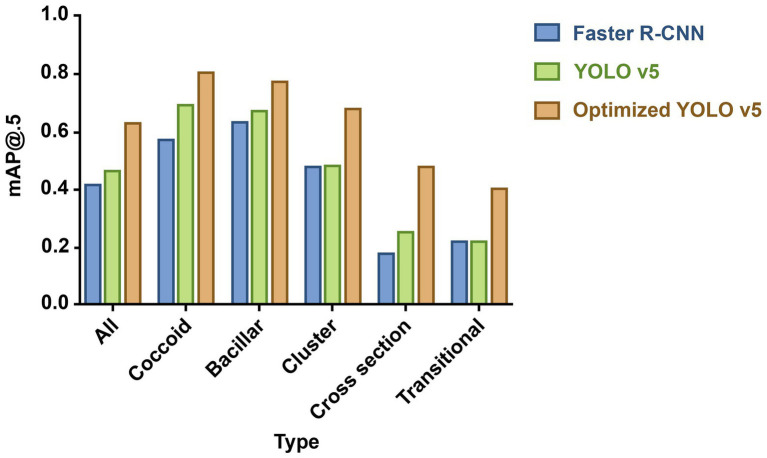
Mean average precision (mAP) for predicting different proposed bacteria detection models. The column height reflects performance: the higher the height, the better the performance. Blue column indicates Faster R-CNN; green column, YOLO v5; and orange column, optimized YOLO v5. And optimized YOLO v5 shows the best performance in detecting various morphological (*H. pylori*).

Two models, Faster R-CNN and YOLO v5, were used to predict the 1,007 sub-images in the test set; the results are shown in Figure 2. The proposed Faster R-CNN and YOLO v5 models achieved an mAP of 0.414 and 0.461, respectively, for detection containing all morphological *H. pylori* types. For coccoid type detection, recall, precision, and mAP values of Faster R-CNN were 0.582, 0.536, and 0.568, respectively; those of YOLO v5 were 0.638, 0.700, and 0.688, respectively. The results showed that the YOLO v5 model offered a better accuracy for HPCF detection than Faster R-CNN, particularly in coccoid detection. Therefore, we chose YOLO v5 as the target model to detect HPCF for further optimization.

### YOLO v5 vs. optimized YOLO v5

Optimized YOLO v5 with data enhancement and model structure optimization achieved an overall mAP value of 0.624; that of YOLO v5 was 0.461 (Figure 2). Optimized YOLO v5 produced better results, attaining a mAP value of 0.803 for coccoid type detection, outperforming YOLO v5 by 16.7%. The different detection models’ specific performance results are shown in Supplementary Appendix S2 and coccoid detection performance results of manual labeling and AI in the test set in Supplementary Appendix S3.

**Figure 3:**
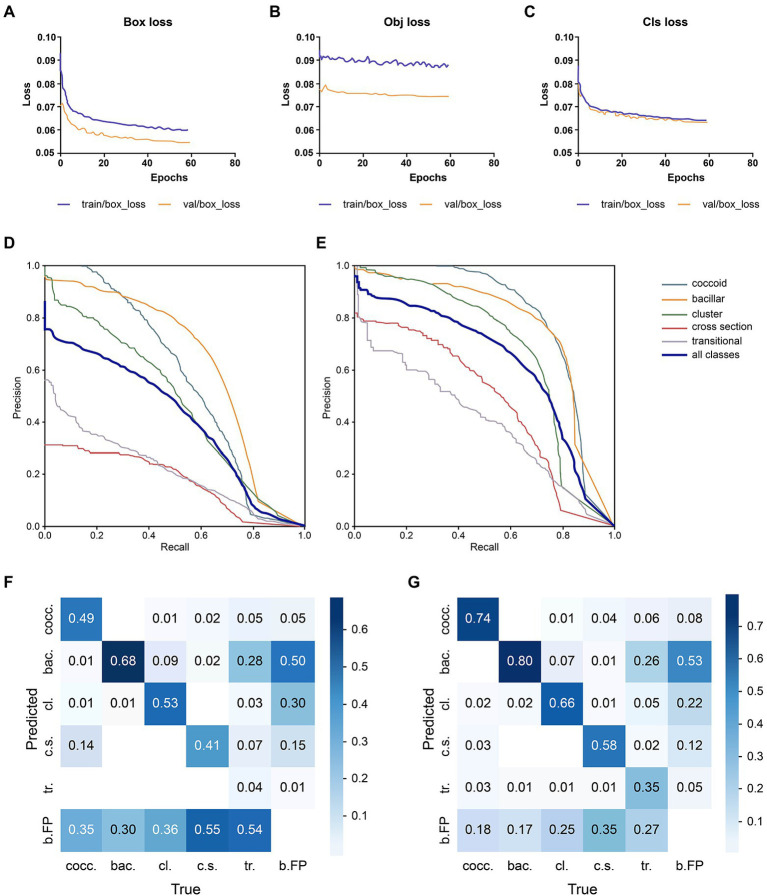
Performance comparison of the deep learning models. **(A)** Bounding box regression loss: assessing the degree of overlap between the prediction and real frames. **(B)** Confidence loss: it calculates whether the grid’s confidence is correct. **(C)** Classification probability loss: assessing the classification. **(D)** YOLO v5 PR curve: it indicates the relationship between precision and recall; rows indicate the recall, whereas columns display the prediction by the model. **(E)** Optimized YOLO v5 PR curve. Optimized YOLO v5’s PR curve **(E)** can completely wrap around YOLO v5’s PR curve **(D)**, indicating that Optimized YOLO v5 outperforms YOLO v5. **(F)** YOLO v5’s confusion matrix. The diagonal values indicate the true positive portion for each object type, and the other values, outside of the diagonal, display the misclassification rates, **(G)** The optimized YOLO v5’s confusion matrix.

**Figure 4:**
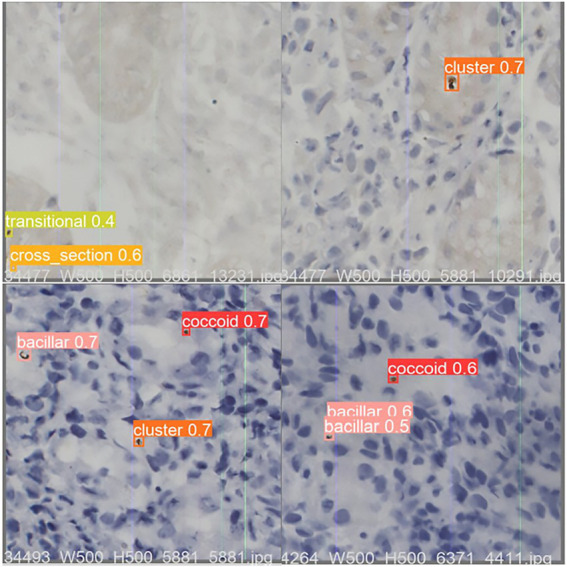
An example of optimized YOLO v5 detection result on test dataset sample images. The value shown in the image is the Intersection over Union (IOU), which measures the correlation between true and predicted as an evaluation function. The IOU is between 0 and 1, with higher values being more relevant. Any prediction above 0.5 is usually considered correct. IOU, area of overlap/area of union.

The loss comparison curve, PR curve, and Confusion matrix are shown in Figure 3. The model converges at 59 epochs, and the loss decreases as the epoch time increases, eventually converging. The bounding box regression (Figure 3A) and training algorithm confidence loss (Figure 3B) values were lower than those of the validation algorithm, and they both had a large drop at the start of training. This means that the model is learning appropriately and efficiently and undergoing gradient descent. For classification probability loss (Figure 3C), the training set and validation set had similar values. The PR curve showed that optimized YOLO v5 (Figure 3E) significantly outperformed YOLO v5 (Figure 3D). A graph of model testing results is shown in Figure 4.

### Manual reading vs. optimized YOLO v5

**Figure 5:**
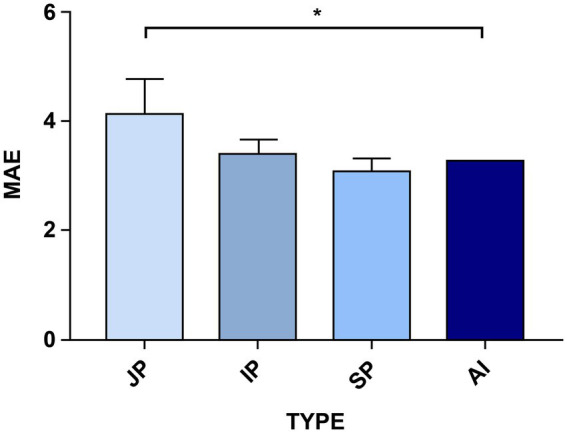
A comparison of the AI models’ and the different-level pathologists’ errors using the Mean Absolute Error (MAE) factor. The optimized YOLO v5 model’s performance was compared with that of nine pathologists with different experience levels who made the test set diagnoses. JP is the junior pathologists, IP is the intermediate pathologists, SP is the senior pathologists, and AI is the improved YOLO v5 detection. The higher the MAE factor value, the worse the error in diagnosing HPCF. The senior pathologists (SP) have the highest detection accuracy for the indices, whereas the model takes second place. Significance is indicated with *p*-value ≤0.05 (*).

To uncover the clinical feasibility and accuracy of the optimized YOLO v5 algorithm for HPCF detection, we compared its coccoid rate detection results with those of manual reading (Supplementary Appendix S4). The coccoid labels’ number and percentage were calculated for each immunohistochemical gastric mucosa sample in the test data. The optimized YOLO v5 and manual reading MAE comparisons are shown in Figure 5. The comparative results showed that, benchmarked against the gold standard, AI model detection had the lowest MAE of 3.25, indicating a minimum error. However, the junior pathologists’ reading had an MAE of 4.14 in their diagnoses compared with the gold standard; that of intermediate pathologists had an MAE of 3.40 and senior practitioners had an MAE of 3.07. This signifies that model detection results are closer to the gold standard, significantly more accurate than those of junior pathologists (3.25 vs. 4.14, *p* < 0.05), no worse than those of intermediate pathologists (3.25 vs. 3.40, *p* > 0.05), and on a par with those of senior pathologists (3.25 vs. 3.07, *p* > 0.05). Moreover, the junior pathologists’ reading results had the largest error compared with the gold standard, although the diagnosis by a senior pathologist was not entirely accurate. This indicates that the AI model has the potential to support more efficient and accurate HPCF diagnosis, replacing junior pathologists and reducing diagnostic errors.

## Discussion

*Helicobacter pylori* coccoid form is an important factor for refractory *H. pylori* infection, the awareness of which helps to eradicate repeated treatment failure (Shah et al., 2021). Under unfavorable and stressful conditions, *H. pylori* shifts from a bacillar form to a coccoid with VBNC state to facilitate survival. The coccoid form of *H. pylori* slows metabolism to facilitate survival, with reduced urease activity, respiratory rate and nutrient uptake, and metabolic protein maintained at basal levels (Fakruddin et al., 2013; Reshetnyak and Reshetnyak, 2017; Kadkhodaei et al., 2020). However, HPCF expresses almost all virulence genes, producing high levels of virulence and oncogenic proteins that may have equivalent or even greater virulence than the bacillar form (Loke et al., 2016). Moreover, HPCF increases the expression of peptidase genes, induces changes in globular cell wall components such as peptidoglycan, or increases the content of cholesterol and unsaturated fatty acids and their accumulation in the cell membrane (Correia et al., 2014; Faghri et al., 2014; Qaria et al., 2018; Brenzinger et al., 2019), enhancing *H. pylori*’s resistance to antibiotics (Brenciaglia et al., 2000). These changes contribute to a better adaptation of *H. pylori* to an unfavorable environment, facilitating its long-term survival in the gastric environment, while also causing it to lose its response to antibiotis and leading to the failure of eradication therapy (Ierardi et al., 2020). Doctors unaware of HPCF will constantly change antibiotics to eradicate *H. pylori*, further aggravating antibiotic resistance and the physical and mental burden on patients. Therefore, in treating refractory *H. pylori* infection, physicians should be aware of the possibility of HPCF, recognize it in time, and stop treatment until the *H. pylori* coccoid form returns to the bacillar form. However, the recognition of HPCF is currently performed by pathologists who read the slides manually, with time-consuming, labor-intensive, subjective variability, and reading limitations and randomness drawbacks.

Rapidly developing AI object detection technology has been successfully applied to many medical issues. Tayebi et al. (2022) applied the YOLO model to automatically identify and detect all bone marrow cells in each region, supporting a more precise hematological diagnosis. Lee et al. (2022) proposed the RCNN model to analyze various abnormal teeth types. Lin et al. (2022) used the residual U-net and V-OMT algorithms to convert irregular 3D brain images into cubes and achieved higher brain tumor detection and segmentation accuracy. The AI model’s object detection may help clinicians make more efficient and accurate diagnoses.

AI object detection technology can be applied to HPCF detection tasks, contributing to solving HPCF detection shortcomings. We selected the classical representative object detection algorithms, YOLO v5 and Faster-RCNN. Faster R-CNN (Ren et al., 2017), one of the best two-stage detectors, generates Region Proposal and utilizes convolutional neural networks to predict the object’s class and location information. YOLO v5, an outstanding representative of the one-stage, extracts features directly from convolutional neural networks to predict the object’s classification and localization. The YOLO v5 model is smaller, with faster training and a shorter inference time than Faster R-CNN (Wu et al., 2021). Moreover, YOLO v5 can assign each bounding box detector to various objects at any possible location in the images, focusing on classifying objects within the bounding box and providing more accurate predictions for individual and smaller objects. YOLO v5 has become the most promising method for object detection, with a fast training phase and superiority over previous YOLO versions (Jocher et al., 2022). We found that the YOLO v5 algorithm (0.688 mAP) is superior to Faster-RCNN (0.568 mAP) in coccoid *H. pylori* detection. Therefore, the YOLO v5 was chosen as our base model for identifying HPCF.

Subsequently, we performed tailored optimization on the YOLO v5 model according to the dataset characteristics with an unbalanced number of labels and a small object size. We chose the Copy-Paste to reduce the influence of imbalanced labels, increasing the amount of scarce coccoid label training data (Ghiasi et al., 2021). Adding CA, multiscale detection, and bounding box anchors parameter optimization; the improved network can extract the detection object’s feature information more effectively, recognizing smaller objects better. The results showed that mAP significantly increased from 0.688 to 0.803 under the same training set, with an improvement of 16.72%.

The comparison between the optimized YOLO v5 model recognition and pathologist’s diagnosis is an important indicator of measuring the model’s clinical evaluation. This reflects the model’s validity, practical significance, and effect.

We concluded that coccoid detection accuracy using the optimized YOLO v5 model (3.25 MAE) is superior to that of junior pathologists (4.14 MAE), not inferior to that of intermediate pathologists (3.40 MAE), and approximately equal to that of experienced senior pathologists (3.07 MAE). The HPCF diagnosis accuracy largely depends on the pathologist’s experience. Therefore, similar morphology that is difficult to distinguish can be misdiagnosed by inexperienced junior pathologists who cannot clearly identify *H. pylori* morphological classifications. Compared with the “gold standard,” where each bacterium is labeled individually, there is a gap in HPCF diagnoses made by senior pathologists, suggesting that the randomly selected field of view interpretation method leads to some errors. The AI diagnostic system can observe complete slices and is not susceptible to external factors, enabling a fairly objective and qualitative pathological diagnosis. The results of this study show that the optimized YOLO v5 model is accurate and fast, reaching the level of senior pathologists, and can assist or replace the pathologist’s manual reading.

## Conclusion

The application of AI models to HPCF diagnosis is of great clinical significance. Owing to the long cycle of training excellent pathologists, the imbalance between the strong demand for diagnosis and the pathologists’ scarce resources has become an important factor limiting HPCF diagnosis development and promotion. AI model accuracy can match that of senior pathologists and reduce the reliance on pathologists’ experience for HPCF diagnosis. Additionally, AI models assist pathologists in HPCF diagnosis. They are accurate, fast, and easy to use, reducing the pathologist’s burden and improving diagnosis efficiency, with a wide range of application prospects. Accurate, fast, and easy-to-use AI diagnostic tools can facilitate more HPCF detection in clinical practice and help physicians make the correct clinical decisions, promoting the development of personalized treatment for refractory *H. pylori* infection.

## Data availability statement

The raw data supporting the conclusions of this article will be made available by the authors, without undue reservation.

## Author contributions

ZZ and HG have made substantial contributions to conception and design. XW has been involved in the acquisition of data and pre-processing. ZZ and JL have been involved in writing the code for the AI algorithm. BZ, LY, and SX have been involved in reading and labeling pathology slides. ZZ and XW have been involved in drafting the manuscript and revising it critically for important intellectual content. HG and GC have given final approval of the version to be published and agreed to be accountable for all aspects of the work in ensuring that questions related to the accuracy or integrity of any part of the work are appropriately investigated and resolved. All authors contributed to the article and approved the submitted version.

## Funding

This work was funded by the Postdoctoral Research Foundation of China (2020M670068ZX), Guangdong Medical Science and Technology Research Fund Project (A2022382), Science and Technology Development Fund of Shanghai Pudong New Area (PKX2021-S08), Xuzhou Science and Technology Bureau Project (KC21169), Xuzhou Health Care Commission Leading Talents (XWRCHT20210), and Jiangsu University, Social Development (JLY2021180).

## Conflict of interest

Author JL was employed by company Unicom Guangdong Industrial Internet Co., Ltd, Guangzhou, China.

The remaining authors declare that the research was conducted in the absence of any commercial or financial relationships that could be construed as a potential conflict of interest.

## Publisher’s note

All claims expressed in this article are solely those of the authors and do not necessarily represent those of their affiliated organizations, or those of the publisher, the editors and the reviewers. Any product that may be evaluated in this article, or claim that may be made by its manufacturer, is not guaranteed or endorsed by the publisher.
