# Supplementary material for: A study on the diagnosis of the Helicobacter pylori coccoid form with artificial intelligence technology
Source: Front Microbiol. 2022 Oct 28;13:1008346. doi: 10.3389/fmicb.2022.1008346 (PMC9651970; doi:10.3389/fmicb.2022.1008346)
Supplement: Supplementary file 1 [file Table_1.DOCX]

**Appendix 1. The label type distribution. (The number of bacillar labels makes up most of the total labels and few coccoid labels, meanings there is an imbalance in the dataset).**


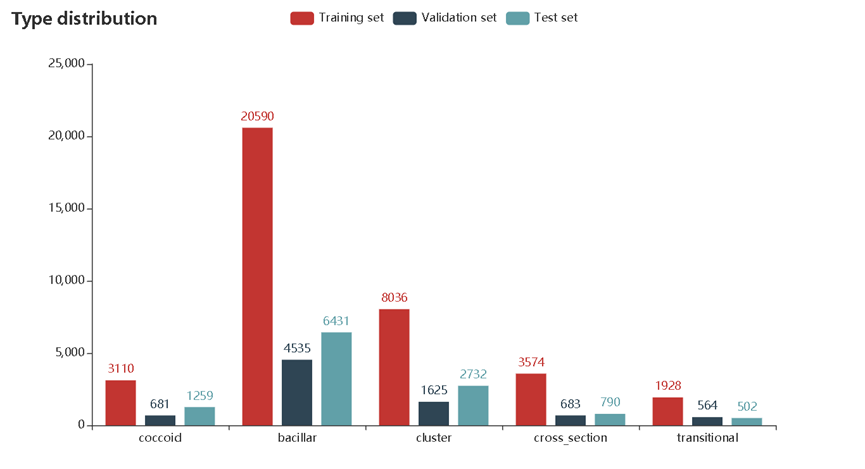


**Appendix 2. Performance of the different detection models (Evaluating the performance of models using precision [Predictive Value]), recall [Sensitivity], and mAP [mean average precision]).**

| **Type** | **Model** | **Labels number** | **Recall** | **Precision** | **mAP** |
| --- | --- | --- | --- | --- | --- |
| **All** | **Faster R-CNN** | 11714 | 0.422 | 0.496 | 0.414 |
|  | **YOLO v5** | 11714 | 0.533 | 0.499 | 0.461 |
|  | **Optimized YOLO v5** | 11714 | 0.626 | 0.696 | 0.624 |
| **Coccoid** | **Faster R-CNN** | 1259 | 0.582 | 0.536 | 0.568 |
|  | **YOLO v5** | 1259 | 0.638 | 0.700 | 0.688 |
|  | **Optimized YOLO v5** | 1259 | 0.740 | 0.800 | 0.803 |
| **Bacillar** | **Faster R-CNN** | 6431 | 0.549 | 0.690 | 0.632 |
|  | **YOLO v5** | 6431 | 0.682 | 0.670 | 0.670 |
|  | **Optimized YOLO v5** | 6431 | 0.800 | 0.480 | 0.769 |
| **Cluster** | **Faster R-CNN** | 2732 | 0.394 | 0.587 | 0.475 |
|  | **YOLO v5** | 2732 | 0.531 | 0.534 | 0.480 |
|  | **Optimized YOLO v5** | 2732 | 0.660 | 0.670 | 0.674 |
| **Cross section** | **Faster R-CNN** | 790 | 0.224 | 0.495 | 0.175 |
|  | **YOLO v5** | 790 | 0.490 | 0.266 | 0.248 |
|  | **Optimized YOLO v5** | 790 | 0.580 | 0.770 | 0.474 |
| **Transitional** | **Faster R-CNN** | 502 | 0.362 | 0.167 | 0.218 |
|  | **YOLO v5** | 502 | 0.324 | 0.323 | 0.217 |
|  | **Optimized YOLO v5** | 502 | 0.350 | 0.760 | 0.399 |

**Appendix 3. Coccoid detection performance results of manual labeling and AI in the test set.**

| Sample ID | All count | Coccoid count | Coccoid percent  (Gold standard) | Coccoid detection count (AI) | Coccoid percent | Accuracy |
| --- | --- | --- | --- | --- | --- | --- |
| 34260 | 2359 | 107 | 4.54% | 69 | 2.92% | 64.32% |
| 34261 | 1139 | 206 | 18.10% | 151 | 13.26% | 73.26% |
| 34264 | 546 | 116 | 21.20% | 104 | 19.05% | 89.86% |
| 34269 | 1512 | 46 | 3.04% | 41 | 2.71% | 89.14% |
| 34271 | 896 | 115 | 12.80% | 50 | 5.58% | 43.59% |
| 34477 | 285 | 47 | 16.50% | 35 | 12.28% | 74.42% |
| 34485 | 791 | 60 | 7.59% | 35 | 4.42% | 58.23% |
| 34493 | 1010 | 136 | 13.50% | 112 | 11.09% | 82.15% |

**Appendix 4. Detailed diagnosis results of different level pathologists on the test set.**

| **Sample ID** | **Senior pathologists** | **Mean** | **Intermediate pathologists** | **Mean** | **Junior pathologists** | **Mean** |
| --- | --- | --- | --- | --- | --- | --- |
| **34260** | 3% | 3% | 2% | 3% | 1% | 2% |
|  | 3% |  | 3% |  | 1% |  |
|  | 3% |  | 1% |  | 4% |  |
| **34261** | 13% | 13% | 11% | 12% | 10% | 10% |
|  | 15% |  | 13% |  | 13% |  |
|  | 11% |  | 12% |  | 7% |  |
| **34264** | 19% | 19% | 15% | 18% | 11% | 15% |
|  | 19% |  | 19% |  | 17% |  |
|  | 19% |  | 20% |  | 17% |  |
| **34269** | 3% | 3% | 3% | 3% | 3% | 2% |
|  | 3% |  | 3% |  | 1% |  |
|  | 3% |  | 3% |  | 2% |  |
| **34271** | 5% | 5% | 5% | 5% | 4% | 4% |
|  | 6% |  | 5% |  | 4% |  |
|  | 4% |  | 5% |  | 4% |  |
| **34477** | 11% | 12% | 10% | 11% | 8% | 10% |
|  | 12% |  | 10% |  | 11% |  |
|  | 13% |  | 13% |  | 11% |  |
| **34485** | 4% | 4% | 3% | 3% | 1% | 3% |
|  | 4% |  | 3% |  | 4% |  |
|  | 4% |  | 3% |  | 4% |  |
| **34493** | 11% | 11% | 13% | 11% | 5% | 10% |
|  | 11% |  | 10% |  | 15% |  |
|  | 11% |  | 10% |  | 10% |  |
